# Achieving Molecular Fluorescent Conversion from Aggregation-Caused Quenching to Aggregation-Induced Emission by Positional Isomerization

**DOI:** 10.3390/molecules27010193

**Published:** 2021-12-29

**Authors:** Xinli Wang, Xiang Lin, Renfu Li, Zexin Wang, Wei Liu, Liwei Chen, Nannan Chen, Tao Dai, Shitao Sun, Zhenli Li, Jinle Hao, Bin Lin, Lijun Xie

**Affiliations:** 1 Department of Oncology, Fujian Medical University Union Hospital, Fuzhou 350001, China; 2 Fujian Provincial Key Laboratory of Screening for Novel Microbial Products, Fujian Institute of Microbiology, Fuzhou 350007, China; acomiy2333@gmail.com (X.L.); Wangzexin1221@126.com (Z.W.); xiguapi20@gmail.com (W.L.); liweichen202112@163.com (L.C.); 13110774889@163.com (N.C.); 3 CAS Key Laboratory of Design and Assembly of Functional Nanostructures, Fujian Key Laboratory of Nanomaterials, Fujian Institute of Research on the Structure of Matter, Chinese Academy of Sciences, Fuzhou 350002, China; lirenfu@fjirsm.ac.cn; 4 State Key Laboratory of Structural Chemistry, Fujian Institute of Research on the Structure of Matter, Chinese Academy of Sciences, Fuzhou 350002, China; daitao@fjirsm.ac.cn; 5 Department of Medicinal Chemistry, School of Pharmaceutical Engineering, Shenyang Pharmaceutical University, Shenyang 110016, China; sunshitao066@163.com (S.S.); l765224871@163.com (Z.L.); 15333570330@163.com (J.H.)

**Keywords:** aggregation-induced emission, mechanofluorochromism, LDs Bioimaging

## Abstract

In this work, we synthesized a pair of positional isomers by attaching a small electron-donating pyrrolidinyl group at ortho- and para-positions of a conjugated core. These isomers exhibited totally different fluorescent properties. PDB2 exhibited obvious aggregation-induced emission properties. In contrast, PDB4 showed the traditional aggregation-caused quenching effect. Their different fluorescent properties were investigated by absorption spectroscopy, fluorescence spectroscopy, density functional theory calculations and single-crystal structural analysis. These results indicated that the substituent position of the pyrrolidinyl groups affects the twisted degree of the isomers, which further induces different molecular packing modes, thus resulting in different fluorescent properties of these two isomers. This molecular design concept provided a new accurate strategy for designing new aggregation-induced emission luminogens.

## 1. Introduction

Organic luminescent materials exhibiting an aggregation-induced emission (AIE) property have attracted a wide range of interest because of their promising applications in fluorescent probes, deformation detectors, security systems, and data storage [1,2,3,4,5,6,7,8]. Therefore, the design and synthesis of materials with AIE-active are of high significance. To date, most AIE-active materials were usually created by combining multiple design strategies, such as π-extended conjugated AIE backbones, the introduction of a donor-acceptor (D-A), the transformation from aggregation-caused quenching (ACQ) to AIE by regioisomerization strategy, and the fusion of AIE-active fluorophores with ACQ fluorophores [9,10,11,12,13,14,15,16,17,18]. Among them, the regioisomerization strategy is becoming one of the most popular ways to construct new aggregation-induced emission luminogens (AIEgens). Normally, this strategy can be achieved by migrating a bulky group to a different position. However, the positional impact of a small group on AIE and mechanofluorochromic (MFC) properties has rarely been further investigated [19,20].

In these previous works, the conversion from ACQ to AIE was realized due to the higher degree of distorted structure by migrating large groups to a different position, and the resulting compounds showed obvious AIE-active optical properties [21,22,23,24]. For example, in 2017 Anupama et al. investigated how triphenylamine (TPA) and tetraphenylethylene (TPE) units have different effects on their AIE and mechanofluorochromic properties at different positions of phenanthroimidazoles. In 2020, Li et al. investigated the migration of TPA from the rotor position of TBP-e-TPA to its bay position to obtain TBP-b-TPA. By changing the position of TPA, two compounds with different optical properties were obtained [25,26]. Therefore, we believe that studying the changes in optical properties caused by the migration of simple units can also achieve a further revelation of the relationship between the structure and AIE activity, which will also provide a guideline for the design of AIE materials’ skeleton.

In this contribution, two new isomers named PDB2 and PDB4 are subtly designed and synthesized. Interestingly, PDB2 exhibits AIE properties, whereas PDB4 shows an ACQ effect. The MFC properties of the two compounds were further investigated due to their different fluorescent properties, and the results demonstrated that PDB2 with an AIE property showed a significant MFC behavior with a multi-stimulus response property, while PDB4 did not possess these properties except for solvatochromism. Furthermore, both the detailed single crystal structure analysis and density functional theory (DFT) theoretical results elucidate that the molecular structure, charge distribution, and packing mode play vital roles in the photophysical processes of two isomers, which helps us understand the influence of the different substituent positions on the AIE phenomenon. This work may provide a new platform for the future design of efficient AIE materials with multi-functional properties, such as mechanochromism, acidochromism, and LDs imaging ability.

## 2. Results and Discussions

### 2.1. Photophysical Properties

**Figure 1:**
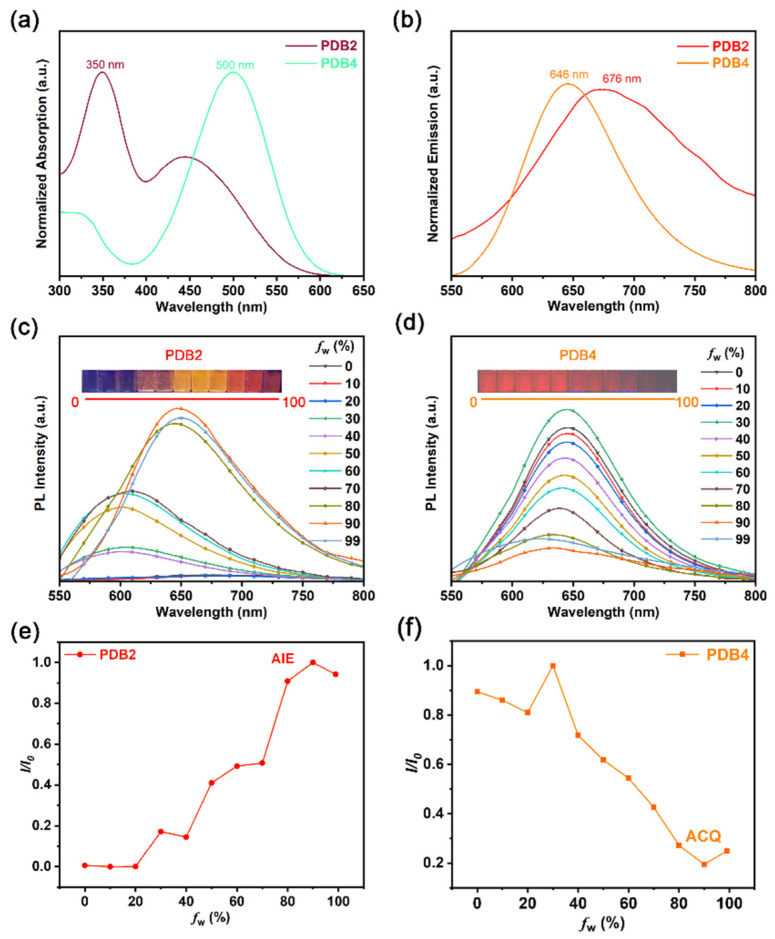
(**a**) UV/vis absorption spectra of PDB2 and PDB4 in DMSO solution (50 μM). (**b**) PL spectra of PDB2 and PDB4 in DMSO solution (50 μM). (**c**,**d**) Emission spectra of PDB2 and PDB4 in water−DMSO mixtures with different water fractions (50 μM). (**e**) Emission intensity changes of PDB2 in different water−DMSO mixture ratios (I_0_: maximal PL intensity of *f*_w_ to 90, I: maximal PL intensity of *f*_w_ to other fraction points). (**f**) Emission intensity changes of PDB4 in different water−DMSO mixture ratios (I_0_: maximal PL intensity of *f*_w_ to 30, I: maximal PL intensity of *f*_w_ to other fraction points).

The UV/vis absorption and emission properties of PDB2 and PDB4 were measured in the dimethyl sulfoxide (DMSO) solution. As shown in Figure 1a, two main absorption bands of PDB2 and PDB4 were observed, respectively. Specifically, the strong absorption band of PDB2 was attributed to spin-allowed π–π* transitions of the compound, and the relatively weak absorption peaks could be assigned to an intramolecular charge transfer (ICT), which was induced by the D-A units, pyrrolidinyl as the electron donor group and cyano as the electron acceptor group. In addition, the intensity of the two absorption bands of PDB4 was opposite to that of PDB2, probably due to the solvatochromic effect [27]. Meanwhile, the emission peak of PDB4 was located at around 646 nm, which was assigned to the ICT emission [28]. When the pyrrolidinyl group was changed from a para-position to ortho-position, the corresponding compound PDB2 exhibited a red-shift and peaked at 676 nm in the emission spectrum (Figure 1b). These results demonstrated that the ICT emission could be controlled well by changing the position of D-A units.

The AIE properties of PDB2 and PDB4 were further investigated by dissolving them in DMSO and then adding water as the poor solvent. As is shown in Figure 1c,d, two molecules displayed emission in DMSO due to the presence of a large π-conjugated structure. Upon enhancing *f*_w_ from 0 to 90%, a significant increase in the PDB2 fluorescence intensity was observed and the maximum emission wavelength of PDB2 changed from 600 nm to 650 nm due to the ICT effect resulting from an increased solvent polarity. With a further increase of *f*_w_ to 99%, the AIE effect diminished because parts of the molecules began to precipitate [29].

In contrast, the fluorescence intensity of PDB4 decreased gradually with the water fraction increasing. The distinct difference in the fluorescence intensity suggested a significant ACQ effect of PDB4 (Figure 1e,f).

### 2.2. Intramolecular Charge Transfer

**Figure 2:**
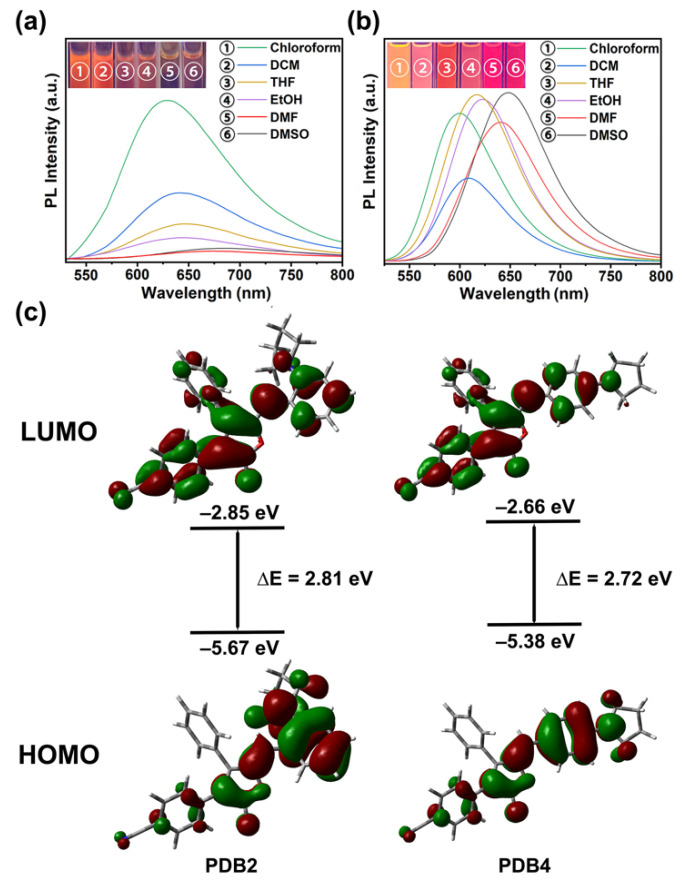
(**a**) Fluorescence spectra of PDB2 (50 μM) in different solvents; (**b**) Fluorescence spectra of PDB4 (50 μM) in different solvents. Inset: fluorescence images (Excited wavelength: 365 nm, 10 mM); (**c**) Calculation of the spatial distribution of HOMO and LUMO for PDB2 and PDB4 in DMSO.

To investigate the ICT effect of PDBs in different solvents with various polarities, we measured the absorption and emission spectra of PDBs in a series of solvents with different polarities. As shown in Figure S7, there was almost no significant change in the absorption spectrum with the change in solvent polarity [30]. On the contrary, by changing the solvent from weakly polar dichloromethane (DCM) to highly polar DMSO, the emission peaks of PDB2 and PDB4 underwent a red shift from 600 to 676 nm and from 602 to 650 nm, respectively, which indicated a typical solvent discoloration effect (Figure 2a,b). At the same time, the fluorescent intensity of PDB2 decreased with the increase of the solvent polarity, which might be due to the positive solvatokinetic effect, while the fluorescence intensity of PDB4 increased with the increase of the solvent polarity, which might be due to the negative lysis-induced kinetic effect. This demonstrated that the ICT effect of PDB2 was stronger than that of PDB4 [29]. Meanwhile, DFT calculations on their energy levels were performed to study the ICT process of the PBDs. The Highest Occupied Molecular Orbital (HOMO) and Lowest Unoccupied Molecular Orbital (LUMO) are shown in Figure 2. The geometry in the PDB2 was more distorted than that of PDB4 (Figure 2c). The HOMO electron cloud of PDB2 was mainly located at the electron donor (i.e., pyrrole group) and its adjacent benzene ring, whereas the LUMO electron cloud of PDB2 was mainly located near the electron acceptor (i.e., cyan group). In contrast, the HOMO electron cloud of PDB4 was mainly delocalized over the conjugated backbone, with a small portion located at the electron donor. In contrast, the LUMO electron cloud of PDB4 was distributed similarly to that of PDB2. Meanwhile, the HOMO-LUMO gaps of PDB2 and PDB4 were 2.81 eV and 2.72 eV, respectively, which indicated that the HOMO and LUMO of these two compounds were separated well and that PDB2 may have a stronger ICT tendency [31,32,33]. We also used the Lippert Mataga plot to interpret solvent-dependent spectral shifts. The Lippert Mataga plot for PDB2 and PDB4 in various solvents is shown in Figure S8 and Table S1. If DCM and ethanol are omitted, the rest of the data show a linear correlation, which suggests that the emission spectra of PDB2 and PDB4 are related to the ICT effect [34].

### 2.3. Crystal Structure

**Figure 3:**
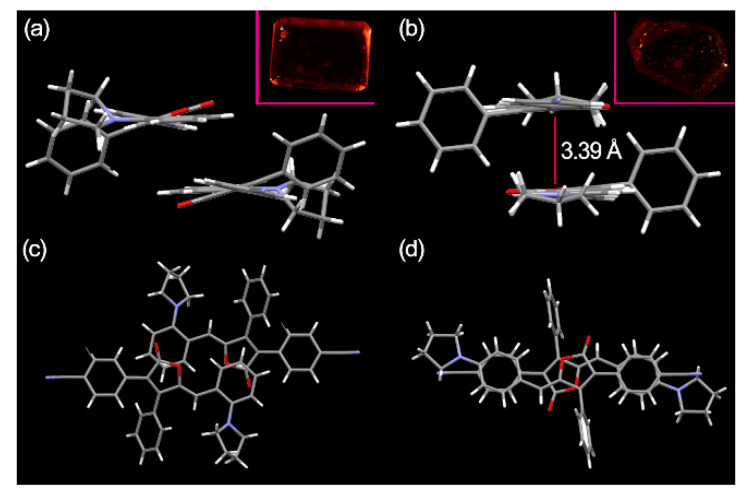
(**a**) Side view of PDB2; (**b**) Side view of PDB4; (**c**) Top view and illustration of no π–π stacking in PDB2; (**d**) Top view and illustration of the π–π stacking in PDB4. Inset: Photos of both the crystal PDB2 and PDB4 under natural light.

The orange PDB2 crystals (CCDC 2102794) and dark red PDB4 crystals (CCDC 2102793) were obtained by slow evaporation of tetrahydrofuran and acetonitrile solution, respectively, and suitable single crystals were selected for single crystal X-ray diffraction analysis (Figure S9). The crystal data are shown in Tables S2 and S3, and the crystal structures of PDB2 and PDB4 are shown in Figure 3. It could be seen that PDB2 was packed more loosely than PDB4 in the crystal without obvious π–π stacking. In PDB2, the lactone ring was packed on top of the hydrogen atom of a benzene ring, not the center of the benzene ring. Such a packing mode decreased the packing interactions significantly. In contrast, in PDB4, the two molecules were packed perfectly on top of each other between these two benzene rings with distance of 3.39 Å, and such π–π interactions contributed greatly to its fluorescent properties.

### 2.4. Mechanofluorochromic Properties

**Figure 4:**
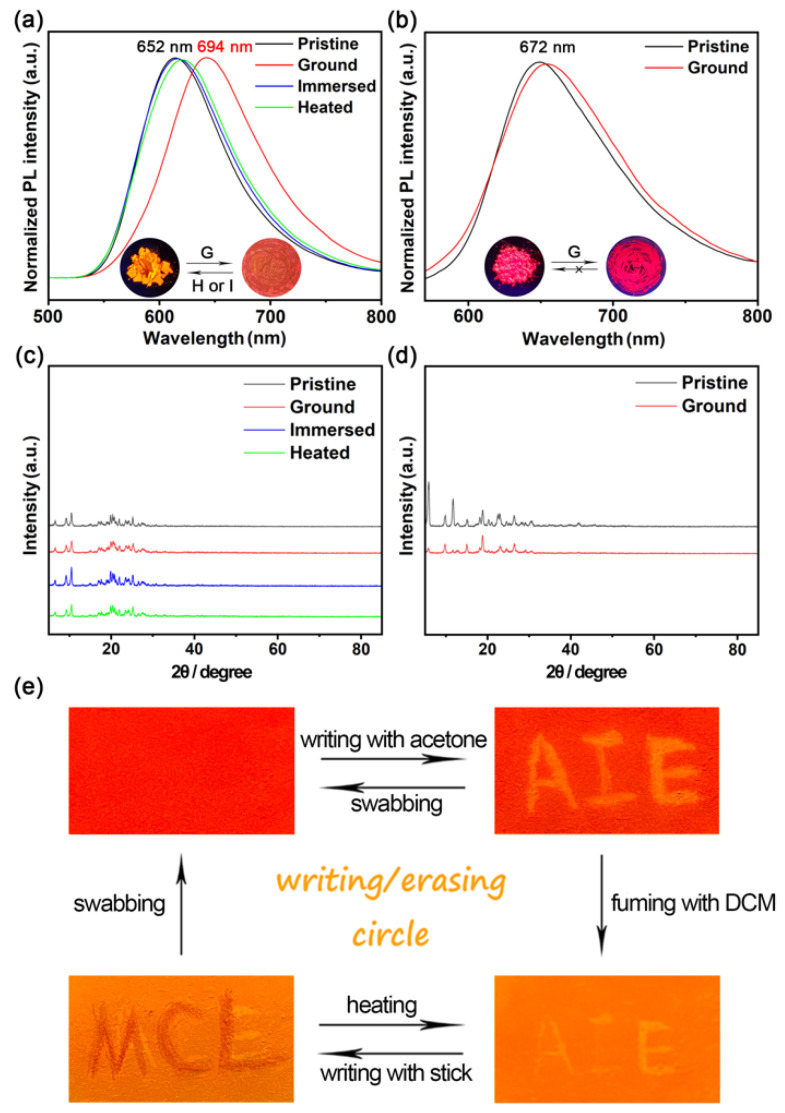
(**a**) Emission spectra of a pristine, ground sample, an immersed sample, and a heated sample of PDB2. (**b**) Emission spectra of a pristine, ground sample of PDB4. (**c**,**d**) Powder X-ray diffraction patterns of PDB2 and PDB4 in different states. (**e**) Photos of PDB2 in the writing/erasing cycle under UV light (λ_ex_ = 365 nm). Inset: Photos upon irradiation with 365 nm UV light of PDB2 and PDB4 in different states.

Recently, AIE-active materials were potential candidates for highly efficient MFC materials. Therefore, taking PDB4 as a control, we investigated the potential MFC properties of PDB2 benefiting from its good AIE properties. Under UV irradiation, the prepared powders PDB2 and PDB4 exhibited orange fluorescence and violet-red fluorescence (Figure S10) with emission peaks of 652 nm and 672 nm, respectively (Figure 4a,b). After the solids of these two isomers were processed by grinding with an agate mortar, an emission redshift of about 42 nm to 694 nm was observed for PDB2. This MFC behavior of PDB2 was reversible, so that the emission color could be recovered and returned to the original emission color by heating the ground sample of PDB2 at 100 °C within 10 min or immersing it in acetone solvent. In contrast, as expected, no MFC phenomenon was observed for the corresponding PDB4. The distinctly opposite MFC phenomena based on these two isomers are worthy of attention in order to investigate the mechanism of the MFC materials.

To further explore the MFC mechanism, the PXRD patterns of the different state powders of PDB2 and PDB4 were obtained. Both pristine powders displayed intense and sharp reflection peaks. This result indicated their well-ordered crystalline nature (Figure 4c,d). On the other hand, there was a small difference in the PXRD of PDB2 in different states, and some of its diffraction curves weakened after grinding, which may be due to the red shift produced by PDB2 and attributed to planarization caused by grinding [29]. However, PDB4 was not an MFC-active material, although its physical phase transformations from a crystalline state to amorphous state happened upon grinding, as shown in Figure 4d [35].

The transition from a crystalline structure to an amorphous state when grinding PDB2 was further confirmed by DSC experiments. The DSC curves of PDB2 (Figure S11) showed an endothermic transition peak at 229 °C. For the ground sample, the endothermic transition peak changed to 228 °C. The endothermic transition peak returned to 229 °C when the ground sample of PDB2 was heated. The endothermic transition peak of the sample immersed in acetone showed no change, but a glass transition appeared at 393.5 °C [36,37,38]. However, the DSC curves of PDB4 did not exhibit properties similar to PDB2 [39]. There was no significant change in the DSC curve of PDB4.

Based on the mechanofluorochromic properties of PDB2, we explored a practical application in a rewritable paper. As shown in Figure 4e, the pristine powder of PDB2 was applied on a piece of weighing paper. Afterwards, the letters were written on the weighing paper with acetone, and the letters emitted yellow light under a 365 nm UV lamp. Then, the fluorescent letters could be easily erased by fuming with DCM for a few minutes; after that, the letters could be rewritten with a rod and wiped off again, and the writing/erasing process could be repeated many times. Therefore, this material shows great potential for applications on rewritable paper [40].

### 2.5. Acidochromism

**Figure 5:**
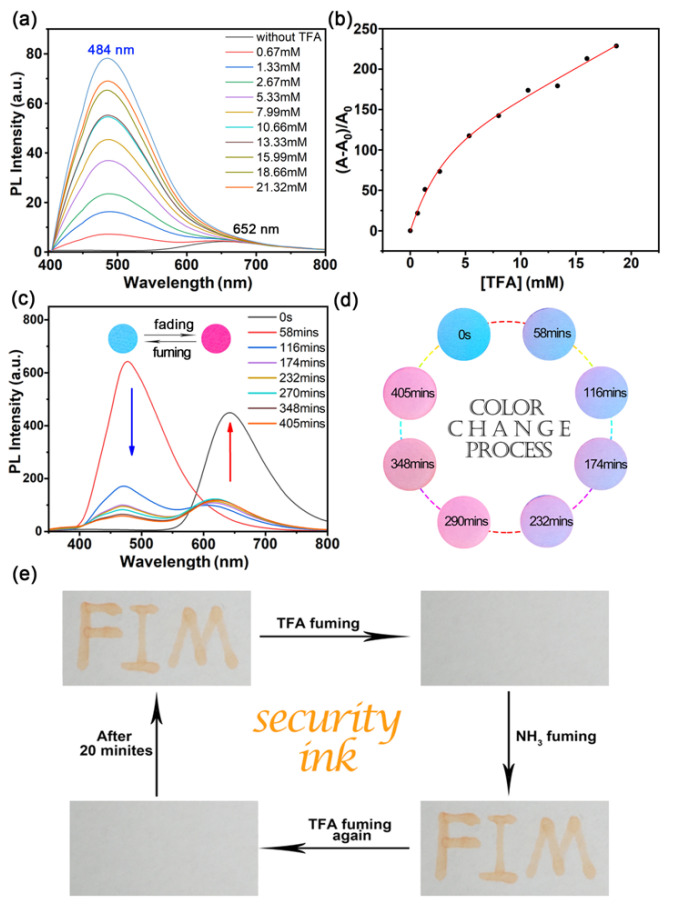
(**a**) Fluorescence spectra of PDB2 (1 × 10^−4^ M) with different concentrations (0.67–21.32 mM) of TFA in the respective solutions of MeOH. (**b**) Fluorescence intensity of PDB2 at 450 nm versus TFA concentration. (**c**) Fluorescence spectra of PDB2 coated on filter paper under a gradual decrease of TFA concentration. (**d**) The color change process of PDB2 filter paper with a gradually decreasing TFA concentration. (**e**) Reversible fluorescence switching by TFA and NH_3_ exposure (λ_ex_ = 365 nm). Inset: Photographs of circular paper sheets treated with dichloromethane PDB2 solution and circular paper sheets after TFA fuming with 365 nm UV light.

As this mechanofluorochromic PDB2 has a basic pyrrolidinyl group, it should be acid-sensitive in a solution or solid state [41]. Therefore, we analyzed the proton-sensing ability of PDB2 as an example. When trifluoroacetic acid (TFA) of different concentrations was added, the absorption band of PDB2 in methanol (MeOH) declined gradually around 410 nm and a new absorption band appeared at 350 nm, which was enhanced when increasing the amount of TFA (Figure S12), and a new blue-shifted peak in the emission spectrum was formed (Figure 5a).

With the concentration of TFA increasing from 0 to 21.32 mM, the PL intensity maximum at 652 nm decreased and a new peak with a PL intensity maximum at 484 nm was formed. This change was due to the characteristic protonation of the pyrrolidine group and the formation of salt [29], while its ratio of fluorescence intensity had a quadratic function in a certain range of TFA concentrations (Figure 5b). However, the formation of this salt was not stable, and the fluorescence intensity of PDB2 was gradually recovered as time passed. Under UV light, we formed a drop of PDB2 in DCM on a piece of filter paper to form a violet-red circle, whose color changed into blue within a few seconds when fuming with TFA vapor; afterwards, the circle returned to violet-red when left at room temperature for some time. This process was further confirmed by the blue shift of the emission spectrum when treated with TFA (Figure 5c,d) [32]. In contrast, PDB4 did not react with trifluoroacetic acid (TFA) to produce these phenomena. The fluorescence of PDB4 was quenched with the addition of different concentrations of TFA, and the solution only emitted a faint red fluorescence under UV light (Figure S13) [33].

The experimental phenomenon of the acidochromic property of PDB2 inspired us to apply this compound in security inks. As depicted in Figure 5e, the letters “FIM” were written with DCM solution of PDB2 (10 mM), and after fuming with TFA for a few seconds, the letters disappeared. Then, the letters would reappear automatically after a while. The letters would also reappear by fuming with ammonia. Moreover, after a few seconds of fuming with TFA, the letters would disappear, and they reappeared after 20 min [42].

### 2.6. Lipid Drops (LDs) Staining Properties

**Figure 6:**
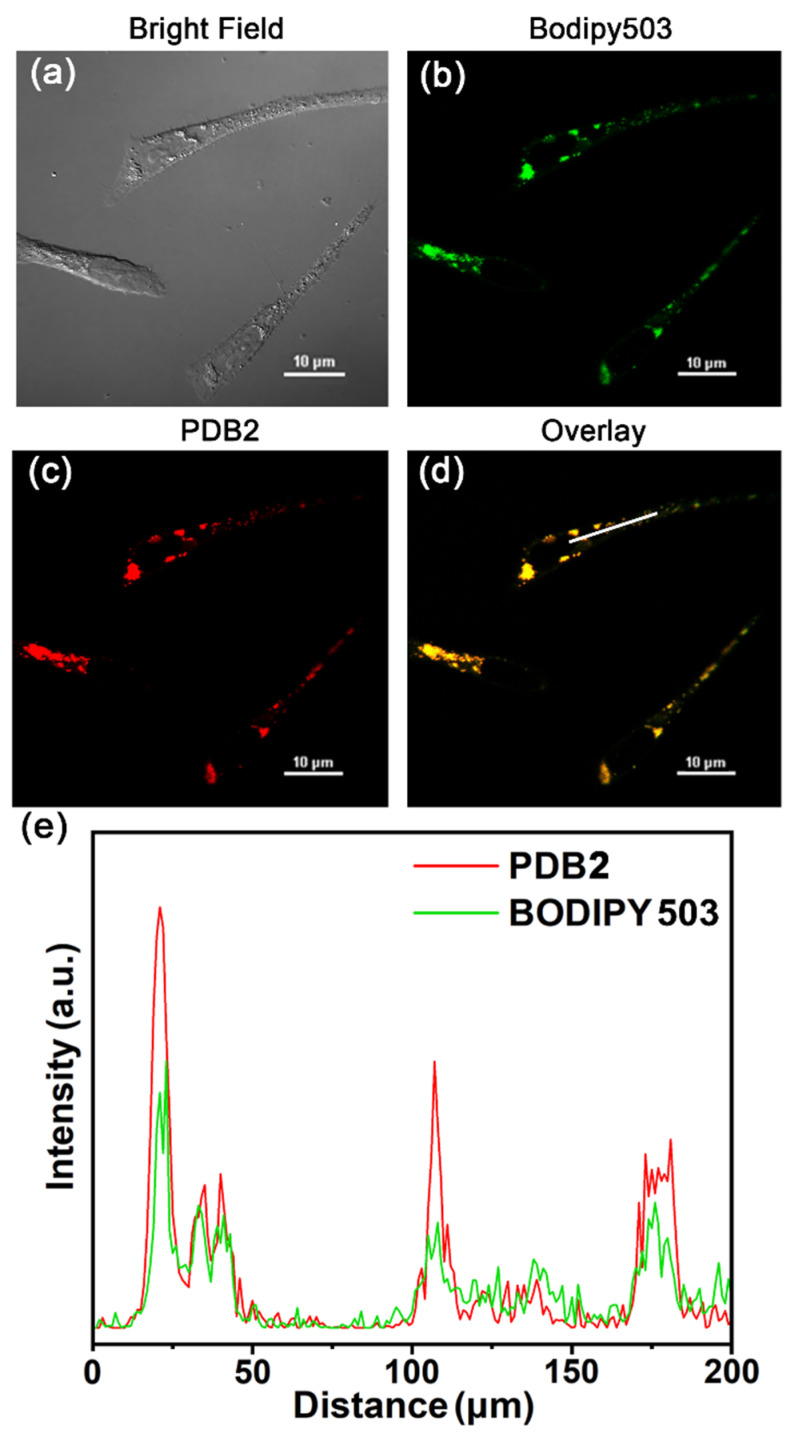
(**a**) Bright-field images of PDB2. (**b**,**c**) CLSM image of HeLa cells with BODIPY 503 (10 μM) and PDB2 (10 μM). (**d**) The merged images with BODIPY503 and PDB2. (**e**) The intensity profile of ROI lines. Scale bar = 200 μM (red and green channel, excitation wavelength, etc.).

We further used PDB2 for the super-resolution imaging of LDs in HeLa cells to investigate the specificity of PDB2 for LDs, and co-localization experiments were performed with the lipid dye BODIPY 503. As shown in Figure 6a, HeLa cells were not stained in the bright field. The LDs in HeLa cells were detected using both PDB2 and BODIPY 503 at a concentration of 10 μM, respectively, to stain HeLa cells. However, at the same concentration, the staining signal-to-noise ratio of PDB2 was significantly greater than that of BODIPY 503 (Figure 6b,c). Additionally, a high overlap of the intensity profiles of PDB2 and BODIPY 503 in the region of interest (ROI) was observed, the intensity changes were closely synchronized, and the fluorescence intensity of PDB2 in the ROI was significantly greater than that of BODIPY 503 in this region (Figure 6d,e). This suggests a better selectivity of PDB2 for LDs. The staining specificity of PDB2 for LDs can be attributed to its lipophilic properties. This allows PDB2 to interact “like-like” with various neutral lipids in hydrophobic spherical LDs, allowing them to accumulate efficiently in hydrophobic spheres and producing high brightness staining properties [43,44].

## 3. Materials and Methods

### 3.1. General Information and Materials

All chemicals were purchased from commercial suppliers without further purification unless otherwise stated. All glassware, needles, and magnetic stirring bars were dried in a vacuum oven. Thin-layer chromatography was used to monitor reactions. ^1^H NMR and ^13^C NMR spectra were collected on Bruker AVANCEIII HD-400. HR-MS data were obtained using Agilent 6545 Q-TOF. Absorption and emission spectra were measured on Thermo Varioskan™ LUX Multifunctional Enzyme Labeler. The powder X-ray diffraction (PXRD) patterns were recorded on a Bruker D8 Quesr/Venture diffractometer with MoKα radiation (λ = 0.77 Å). The differential scanning calorimetry (DSC) curves were determined using a heat flow DSC. The crystal structures of PDB2 and PDB4 were solved using direct methods and then refined by the full-matrix least squares refinements on F2 using the SHELXLTL software package. The absolute photoluminescence quantum yields (PLQYs) of the samples were obtained by employing a standard barium sulfate coated integrating sphere (150 mm in diameter, Edinburgh) as the sample chamber, which was mounted on the FLS980 spectrometer with the entry and output ports of the sphere located at a 90° geometry from each other in the plane of the spectrometer. A standard tungsten lamp was used to correct the optical response of the instrument. All the spectral data were collected at RT and corrected for the spectral response of both the spectrometer and the integrating sphere. Lipid droplets (LDs) imaging was performed with a Nikon Ti-E&C2 scanning unit.

### 3.2. Synthesis

**Scheme 1:**
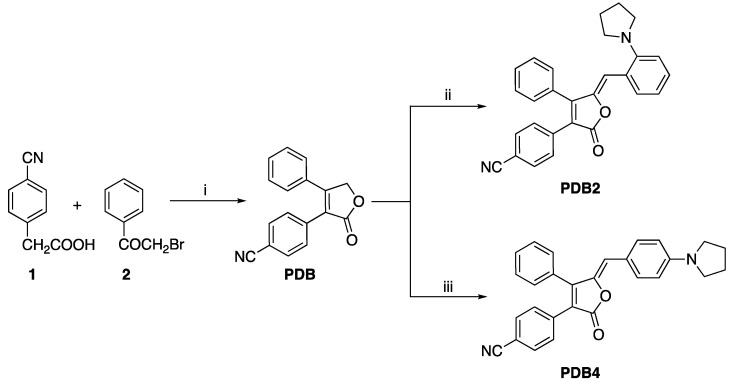
Synthetic routes for PDB2 and PDB4. Reaction condition: (i) Et_3_N, 25 °C, 30 min; DBU, −5 °C, 20 min. (ii) 2-(1-Pyrrolidinyl) benzaldehyde, piperidine, 25 °C, 24 h. (iii) 4-(1-Pyrrolidinyl) benzaldehyde, piperidine, 25 °C, 24 h.

The synthetic routes of PDB2 and PDB4 are shown in Scheme 1.

PDB2 and PDB4 were obtained via a two-step procedure. First, 4-cyanophenylacetic acid (2.50 g, 0.015 mol) and 2-bromoacetophenone (3.08 g, 0.015 mol) were dissolved in acetonitrile solution (65 mL), and then triethylamine (5.07 mL) and DBU (1.81 mL) were added as catalysts to obtain PDB. Then, PDB was dissolved with two different benzaldehydes (0.14 g, 0.798 mmol) in methanol (10 mL), and two drops of piperidine (0.1 mL) were added to obtain PDB2 and PDB4**,** respectively. All the compounds were characterized by ^1^H NMR, ^13^C NMR, HR-MS, and single crystal X-ray data. The relevant data were collected from the original spectra and are listed in the Supplementary Materials (Figures S1–S6).

## 4. Conclusions

In summary, a new strategy for achieving the conversion from ACQ to AIE has been realized by shifting the position of a small pyrrolidinyl group on the benzene ring, which is different from traditional molecular design philosophy relying on the migration of bulky groups. This work provides new space to develop and diversify the library of AIEgens without destroying the π conjugation system. Consequently, a new NIR AIEgens was successfully developed from these two isomers. Furthermore, the single-crystal structure analysis and DFT calculations data clearly elucidate the nature of the AIE and MFC phenomena of PDB2 at the molecular level. The AIE-active PDB2 shows a discrete cross packing mode without π–π stacking, while the ACQ-dominated PDB4 adopts a long-range molecular packing mode with obvious π–π stacking. Moreover, the PXRD, DSC study of these two isomers reveals that the similar physical phase transformations from a crystalline state to amorphous state were responsible for the extremely opposite MFC properties. In a further biological experiment, PDB2 shows a strong LDs imaging ability and high selectivity. This work might provide particularly valuable information for the further understanding of the AIE mechanism and supply new guidelines for the design and preparation of new promising AIEgens with multi-functional properties.

## Data Availability

Not applicable.

## Supplementary Materials

The following supporting information can be downloaded at, the supporting information includes synthesis routes, NMR data and MS spectra, crystallographic data, and additional data of the compounds PDB2 and PDB4.

## Sample Availability

Samples of the compounds PDB2 and PDB4 are available from the authors.
