# Supplementary material for: Achieving Molecular Fluorescent Conversion from Aggregation-Caused Quenching to Aggregation-Induced Emission by Positional Isomerization
Source: Molecules. 2021 Dec 29;27(1):193. doi: 10.3390/molecules27010193 (PMC8747061; doi:10.3390/molecules27010193)
Supplement: Supplementary file 1 [file molecules-27-00193-s001.zip › molecules-1522679-supplementary.pdf]

Achieving Molecular Fluorescent Conversion from Aggregation-Caused Quenching to Aggregation-Induced Emission by Positional Isomerization

Xinli Wang [1, #, *], Xiang Lin [2, #], Renfu Li [3], Zexin Wang [2], Wei Liu [2], Liwei Chen [2], Nannan Chen [2], Tao Dai [5], Shitao Sun [4], Zhenli Li [4], Jinle Hao [4], Bin Lin [4, *] and Lijun Xie [2, *]

1. Department of Oncology, Fujian Medical University Union Hospital, Fuzhou, Fujian 350007, PR China.

2. Fujian Provincial Key Laboratory of Screening for Novel Microbial Products, Fujian Institute of Microbiology, Fuzhou, Fujian 350007, PR China.

3. CAS Key Laboratory of Design and Assembly of Functional Nanostructures, and Fujian Key Laboratory of Nanomaterials, Fujian Institute of Research on the Structure of Matter, Chinese Academy of Sciences, Fuzhou, Fujian 350002, PR China.

4. Department of Medicinal Chemistry, School of Pharmaceutical Engineering, Shenyang Pharmaceutical University, Shenyang, Liaoning 110016, PR China.

5. Fujian Institute of Research on the Structure of Matter, Chinese Academy of Sciences, Fuzhou, Fujian 350002, PR China.

Table of Contents

Section	Title	Page
Figure S1–S6	Characterization of compounds	S3
Figure S7	Spectra data of compounds in different solvents	S9
Table S1	The photophysical data of **PDB2** and **PDB4** in different solvents.	S9
Figure S8	Lippert Mataga plot	S9
Figure S9	Molecular conformation of **PDB2** and **PDB4**	S10
Table S2	Dihedral angles of PDB2 and PDB4	S10
Table S3	Single crystal structure data of PDB2 and PDB4	S10
Figure S10	Photographs of solid compounds	S10
Figure S11	DSC curves of PDB2 and PDB4	S11
Figure S12	UV/vis absorption spectra of PDB2 with TFA	S11
Figure S13	Fluorescence spectra of PDB4 with TFA	S12

Characterization of PDB

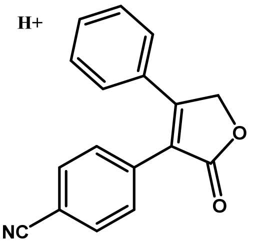

^{1}H NMR (400 MHz, $CDCl_3$, 19.9℃) δ 7.65 (d, *J* = 8.6 Hz, 2H), 7.56 (d, *J* = 8.6 Hz, 2H), 7.48 – 7.42 (m, 1H), 7.41 – 7.33 (m, 2H), 7.28 – 7.23 (m, 2H), 5.20 (s, 2H).

^{13}C NMR (101 MHz, $CDCl_3$, 19.9℃) δ 179.1, 158.9, 135.0, 132.5, 132.5, 132.5, 131.4, 130.2, 129.5, 129.5, 129.5, 127.6, 127.6, 127.6, 124.5, 118.6, 112.6, 70.9.

HRMS–ESI (m/z): [M+H] $^{+}$ calcd for $C_{17}H_{11}NO_2^{+}$, 262.0863; found, 261.0858.

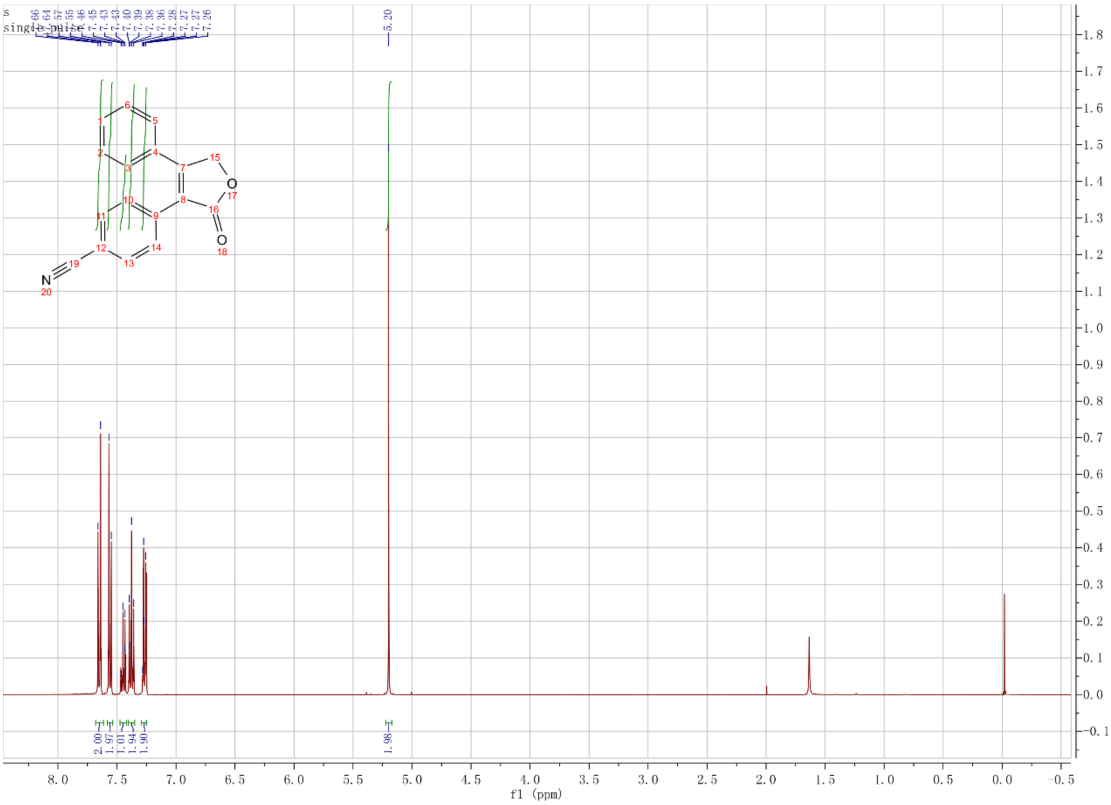

Figure S1. ^{1}H NMR spectrum of **PDB** in $CDCl_3$.

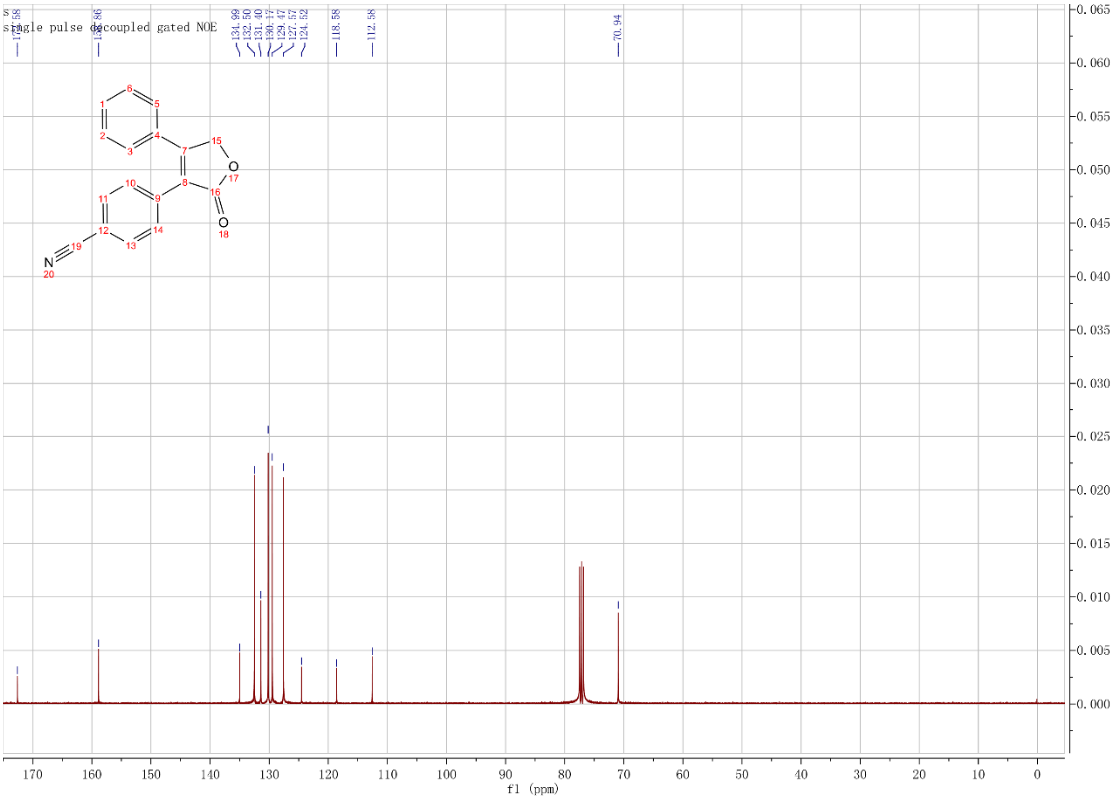

Figure S2. ^{13}C NMR spectrum of **PDB** in $CDCl_3$.

Characterization of PDB2

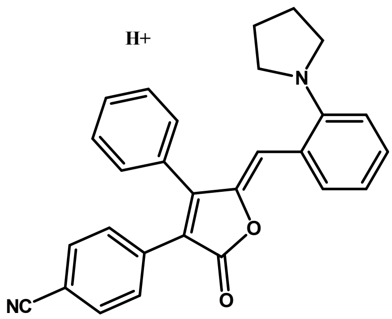

^{1}H NMR (400 MHz, $CDCl_3$, 19.9℃) δ 8.09 (d, J = 7.9 Hz, 1H), 7.57 (d, J = 8.7 Hz, 2H), 7.55 – 7.45 (m, 5H), 7.32 (d, J = 8.0 Hz, 2H), 6.96 (t, J = 7.5 Hz, 1H), 6.88 (d, J = 7.8 Hz, 1H), 6.36 (s, 1H), 3.11 (s, 4H), 1.81 (t, J = 6.3 Hz, 4H).

^{13}C NMR (101 MHz, $CDCl_3$, 19.9℃) δ 171.5, 168.4, 153.0, 146.4, 134.5, 132.3, 132.1, 130.5, 130.3, 130.1, 129.6, 129.6, 129.6, 129.4, 128.9, 128.9, 118.7, 111.8, 52.8, 25.1, 0.1.

HRMS–ESI (m/z): $[M+H]^+$ calcd for $C_{28}H_{23}N_2O_2^+$, 419.1754; found, 419.1764.

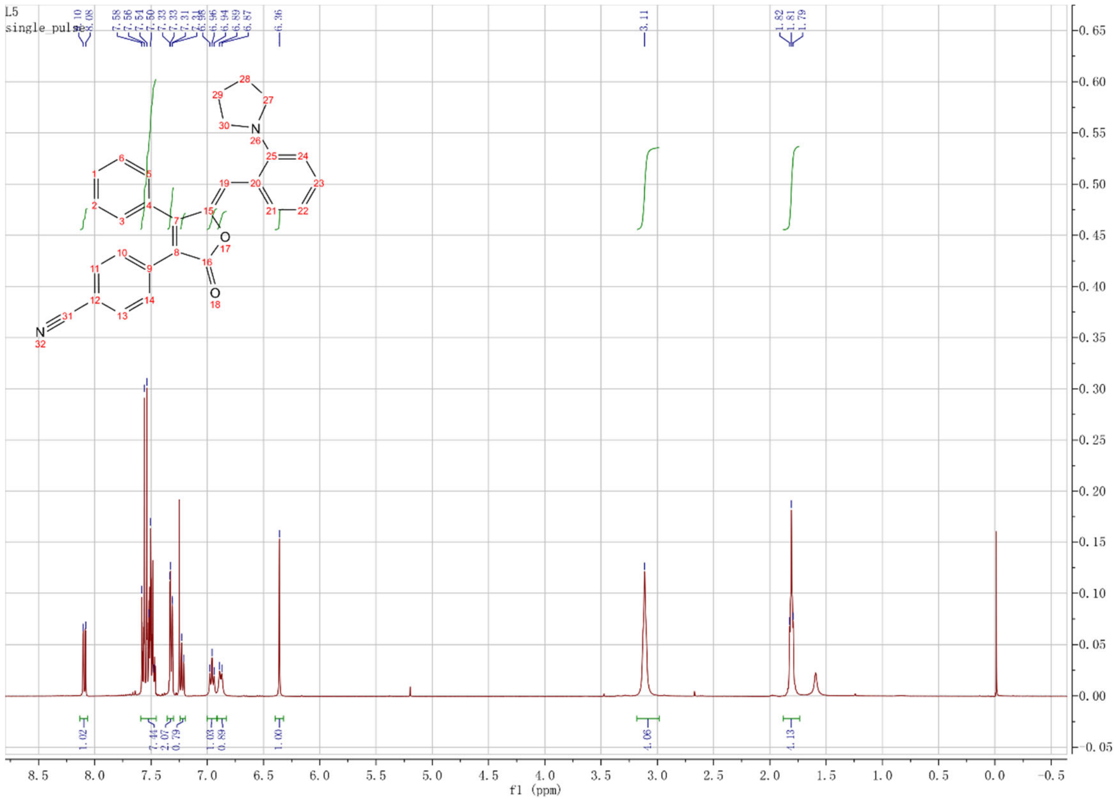

Figure S3. ^{1}H NMR spectrum of **PDB2** in $CDCl_3$.

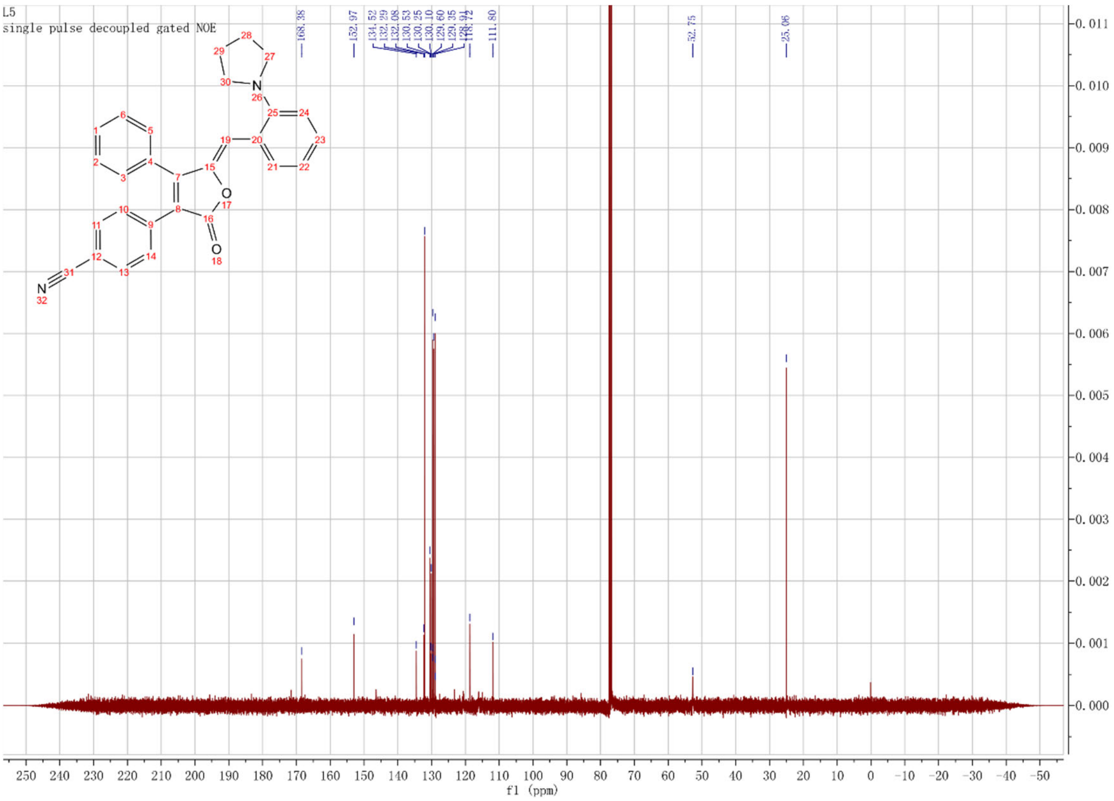

Figure S4. ^{13}C NMR spectrum of **PDB2** in $CDCl_3$.

Characterization of PDB4

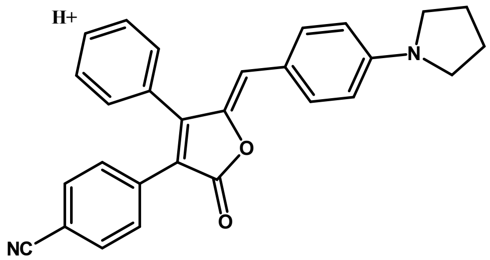

^{1}H NMR (400 MHz, $CDCl_3$, 19.9℃) δ 7.70 (d, *J* = 9.2 Hz, 1H), 7.56 – 7.44 (m, 8H), 7.34 – 7.28 (m, 1H), 6.54 (d, *J* = 9.3 Hz, 1H), 5.99 (s, 1H), 3.40 – 3.31 (m, 4H), 2.08 – 1.98 (m, 4H).

^{13}C NMR (101 MHz, $CDCl_3$, 19.9℃) δ 168.7, 152.6, 148.8, 144.7, 135.2, 133.4, 132.0, 132.0, 130.7, 129.8, 129.4, 129.3, 129.3, 129.3, 129.1, 120.7, 119.4, 118.9, 117.7, 112.1, 111.1, 47.8, 25.5.

HRMS–ESI (m/z): $[M+H]^+$ calcd for $C_{28}H_{22}N_2O_2^+$, 419.1754; found, 419.1757.

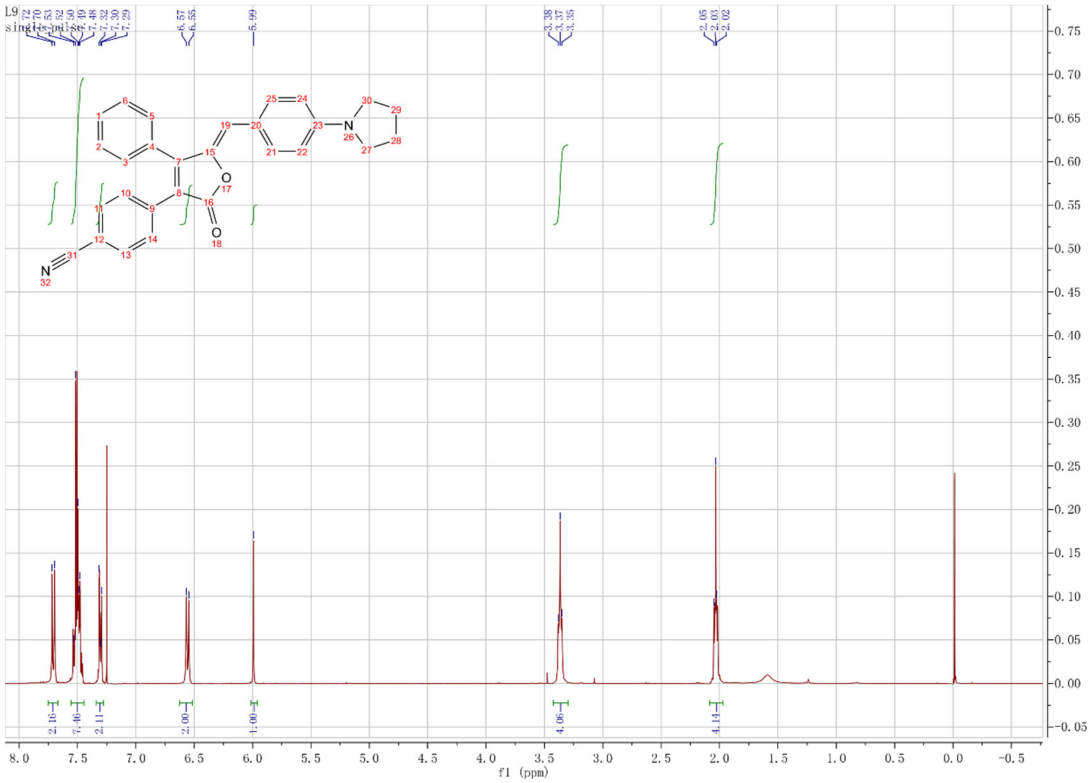

Figure S5. ^{1}H NMR spectrum of **PDB4** in $CDCl_3$.

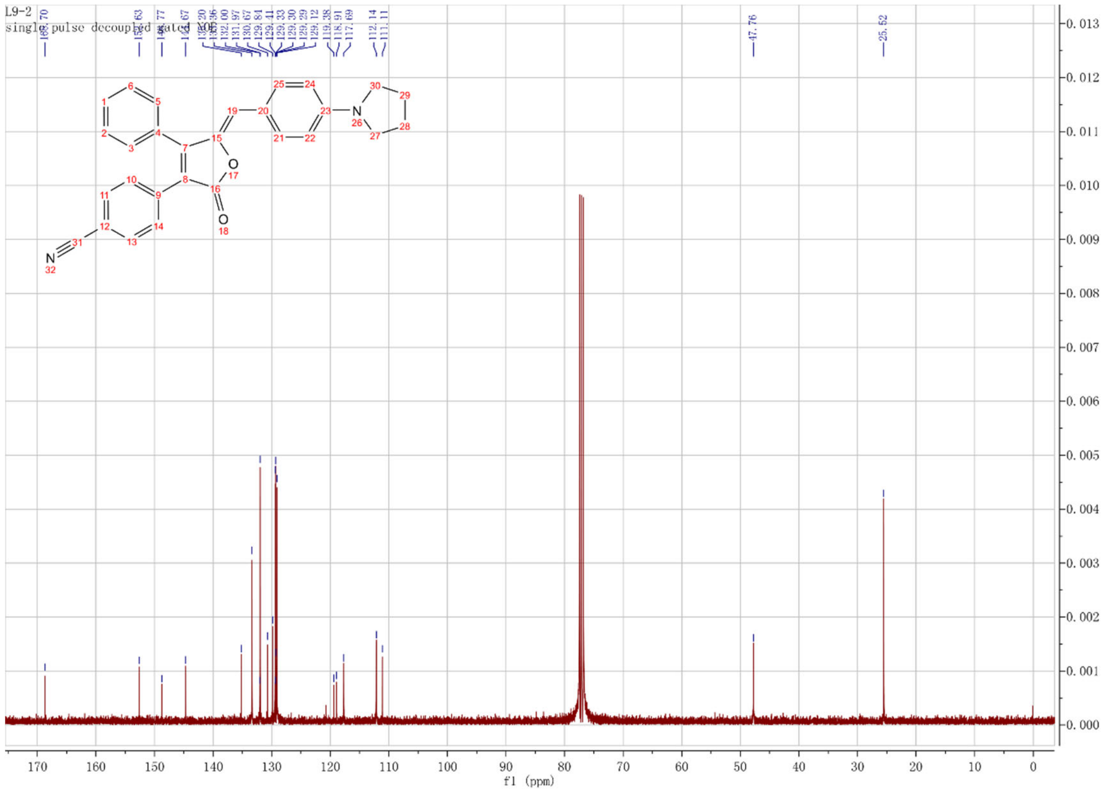

Figure S6. ^{13}C NMR spectrum of **PDB4** in $CDCl_3$.

Spectra data of compounds in different solvents

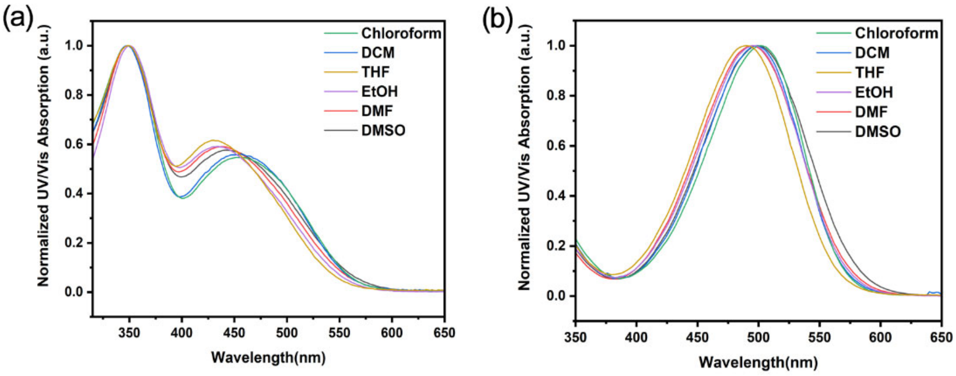

Figure S7. UV−vis spectra of **PDB2** (a) and **PDB4** (b) (5×10^{-5}mol/L) in in different solvents.

Table S1. The photophysical data of **PDB2** and **PDB4** in different solvents.

Compounds	Solvents	λ_{abs} (max) / nm	λ_{em} (max) / nm	Stokes shift / cm^{-1}
**PDB2**	Chloroform	348	630	12862
	DCM	348	645	13231
	Tetrahydrofuran (THF)	348	650	13351
	Ethanol (EtOH)	348	648	13303
	N, N-Dimethylformamide (DMF)	350	676	13942
	DMSO	348	680	14029
**PDB4**	Chloroform	502	600	3253
	DCM	498	604	3524
	Tetrahydrofuran (THF)	490	614	4121
	Ethanol (EtOH)	496	622	4084
	N, N-Dimethylformamide (DMF)	494	642	4666
	DMSO	500	650	4615

Lippert Mataga plot

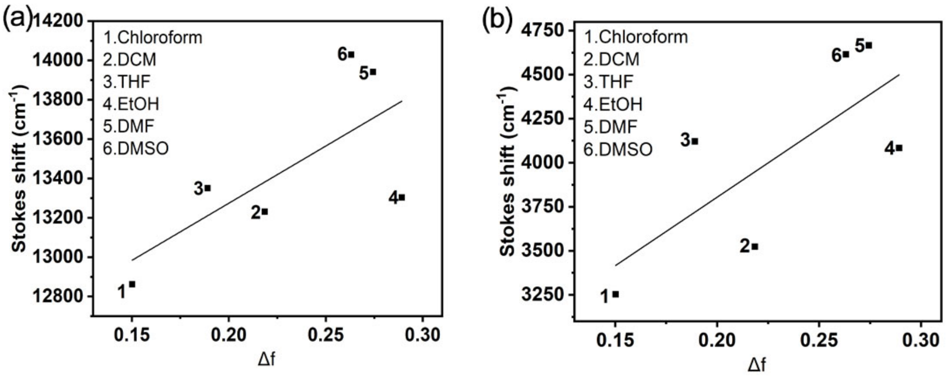

Figure S8. Lippert Mataga plot for **PDB2** (a) and **PDB4** (b) in different solvents showing the variation of Stokes' shift as a function of orientation polarisability of the solvents.

Molecular conformation of PDB2 and PDB4

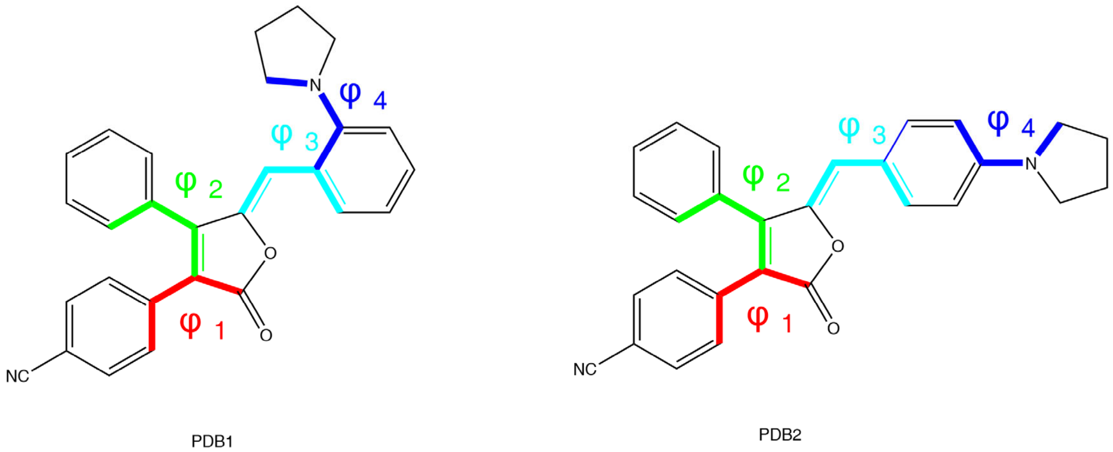

Figure S9. Molecular conformation of **PDB2** and **PDB4** in single crystals, respectively.

Dihedral angles of PDB2 and PDB4

Table S2. Dihedral angles of **PDB2** and **PDB4**

	Dihedral angles (°)			
	θ_{red}	θ_{green}	θ_{blue}	θ_{indigo}
PDB1	30.84	53.47	-16.90	-39.66
PDB2	-6.35	-81.88	-3.43	1.02

Single crystal structure data of PDB2 and PDB4

Table S3. Single crystal structure data of **PDB2** and **PDB4**

	Space Group	Cell Lengths			Cell Angles			Cell Volume
		a	b	c	α	β	γ	
PDB1	P1 bar (2)	9.4951	9.9874	26.3220	91.266	91.736	90.303	2494.35
PDB2	$P2_1/c$ (14)	15.9028	7.7143	18.677	90	108.312	90	2175.24

Photographs of solid compounds

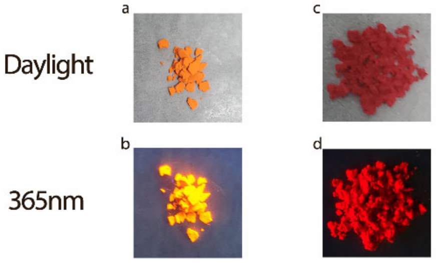

Figure S10. PDB2 solid powder (a, b) and **PDB4** solid powder (c, d) under different normal light and UV light respectively.

DSC curves of PDB2 and PDB4

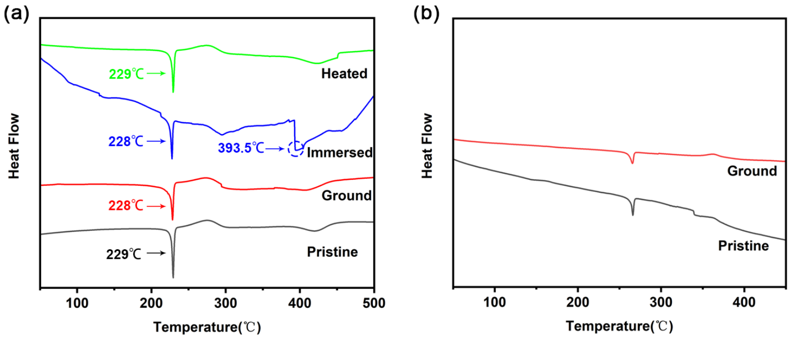

Figure S11. (a) DSC curves of **PDB2** in different states. (b) DSC curves of **PDB4** in different states.

UV/vis absorption spectra of PDB2 with TFA

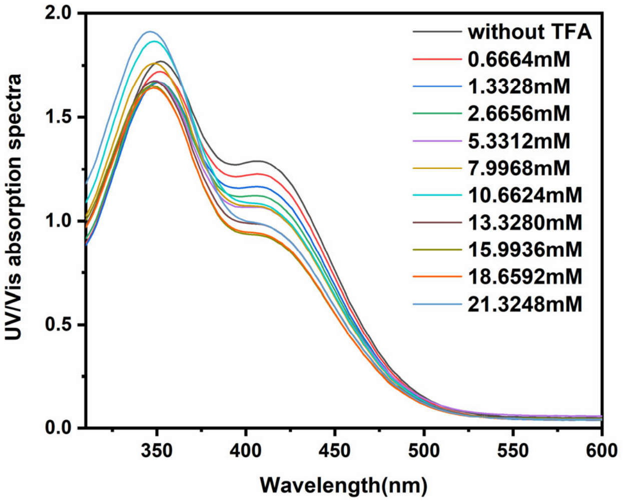

Figure S12. UV/vis absorption spectra of **PDB2** with different concentration (0 – 21.3248 M) of TFA in the respective solutions of MeOH.

Fluorescence spectra of PDB4 with TFA

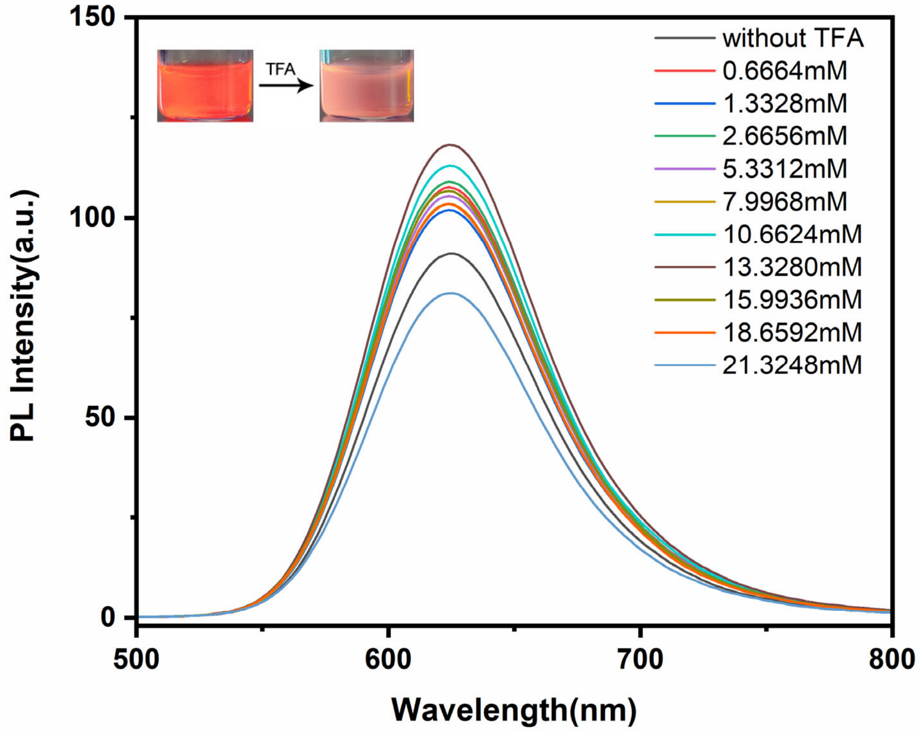

Figure S13. Fluorescence spectra of **PDB4** (1×10^{-4} M) with different concentration (0 – 21.3248 M) of TFA in the respective solutions of MeOH.
